# The Effects of Trunk Muscle Training on Physical Fitness and Sport-Specific Performance in Young and Adult Athletes: A Systematic Review and Meta-Analysis

**DOI:** 10.1007/s40279-021-01637-0

**Published:** 2022-01-21

**Authors:** Atle H. Saeterbakken, Nicolay Stien, Vidar Andersen, Suzanne Scott, Kristoffer T. Cumming, David G. Behm, Urs Granacher, Olaf Prieske

**Affiliations:** 1 Department of Sport, Food and Natural Sciences, Faculty of Education, Arts and Sports, Western Norway University of Applied Sciences, Campus Sogndal, Røyrgata 6, 6856 Sogndal, Norway; 2 Department of Sport and Health Sciences, University of Exeter, Exeter, UK; 3 Faculty of Health and Welfare, Østfold University College, Halden, Norway; 4 School of Human Kinetics and Recreation, Memorial University of Newfoundland, St. John’s, NL Canada; 5 Division of Training and Movement Sciences, Research Focus Cognitive Sciences, University of Potsdam, Potsdam, Germany; 6 Division of Exercise and Movement, University of Applied Sciences for Sports and Management Potsdam, Potsdam, Germany

## Abstract

**Background:**

The role of trunk muscle training (TMT) for physical fitness (e.g., muscle power) and sport-specific performance measures (e.g., swimming time) in athletic populations has been extensively examined over the last decades. However, a recent systematic review and meta-analysis on the effects of TMT on measures of physical fitness and sport-specific performance in young and adult athletes is lacking.

**Objective:**

To aggregate the effects of TMT on measures of physical fitness and sport-specific performance in young and adult athletes and identify potential subject-related moderator variables (e.g., age, sex, expertise level) and training-related programming parameters (e.g., frequency, study length, session duration, and number of training sessions) for TMT effects.

**Data Sources:**

A systematic literature search was conducted with PubMed, Web of Science, and SPORTDiscus, with no date restrictions, up to June 2021.

**Study Eligibility Criteria:**

Only controlled trials with baseline and follow-up measures were included if they examined the effects of TMT on at least one measure of physical fitness (e.g., maximal muscle strength, change-of-direction speed (CODS)/agility, linear sprint speed) and sport-specific performance (e.g., throwing velocity, swimming time) in young or adult competitive athletes at a regional, national, or international level. The expertise level was classified as either elite (competing at national and/or international level) or regional (i.e., recreational and sub-elite).

**Study Appraisal and Synthesis Methods:**

The methodological quality of TMT studies was assessed using the Physiotherapy Evidence Database (PEDro) scale. A random-effects model was used to calculate weighted standardized mean differences (SMDs) between intervention and active control groups. Additionally, univariate sub-group analyses were independently computed for subject-related moderator variables and training-related programming parameters.

**Results:**

Overall, 31 studies with 693 participants aged 11–37 years were eligible for inclusion. The methodological quality of the included studies was 5 on the PEDro scale. In terms of physical fitness, there were significant, small-to-large effects of TMT on maximal muscle strength (SMD = 0.39), local muscular endurance (SMD = 1.29), lower limb muscle power (SMD = 0.30), linear sprint speed (SMD = 0.66), and CODS/agility (SMD = 0.70). Furthermore, a significant and moderate TMT effect was found for sport-specific performance (SMD = 0.64). Univariate sub-group analyses for subject-related moderator variables revealed significant effects of age on CODS/agility (*p* = 0.04), with significantly large effects for children (SMD = 1.53, *p* = 0.002). Further, there was a significant effect of number of training sessions on muscle power and linear sprint speed (*p* ≤ 0.03), with significant, small-to-large effects of TMT for > 18 sessions compared to ≤ 18 sessions (0.45 ≤ SMD ≤ 0.84, *p* ≤ 0.003). Additionally, session duration significantly modulated TMT effects on linear sprint speed, CODS/agility, and sport-specific performance (*p* ≤ 0.05). TMT with session durations ≤ 30 min resulted in significant, large effects on linear sprint speed and CODS/agility (1.66 ≤ SMD ≤ 2.42, *p* ≤ 0.002), whereas session durations > 30 min resulted in significant, large effects on sport-specific performance (SMD = 1.22, *p* = 0.008).

**Conclusions:**

Our findings indicate that TMT is an effective means to improve selected measures of physical fitness and sport-specific performance in young and adult athletes. Independent sub-group analyses suggest that TMT has the potential to improve CODS/agility, but only in children. Additionally, more (> 18) and/or shorter duration (≤ 30 min) TMT sessions appear to be more effective for improving lower limb muscle power, linear sprint speed, and CODS/agility in young or adult competitive athletes.

**Supplementary Information:**

The online version contains supplementary material available at 10.1007/s40279-021-01637-0.

## Key Points

**Table:** 

This meta-analysis investigated the effects of trunk muscle training (TMT) on physical fitness (e.g., maximal muscle strength, CODS/agility, linear sprint speed) and sport-specific performance (e.g., throwing velocity, drive distance, swimming time) in apparently healthy, competitive athletes.
Overall, our analyses showed small-to-large effects of TMT on physical fitness and moderate effects on sport-specific performance in favor of TMT, when compared to active controls.
Participants’ age significantly modulated on CODS/agility with a positive effect of TMT in children.
In terms of training-related programming parameters, a larger effect of TMT on physical fitness was found for higher volume (> 18 sessions) and shorter duration (≤ 30 min) of sessions.

## Introduction

Exercise protocols specifically targeting trunk muscles have been applied for both prevention and rehabilitation of low back pain [1, 2], and the effects of this approach have been examined in several reviews over recent years [3–5]. Whereas initial regimens identified intra-pelvic, spine, and anterior abdominal muscles, that were proposed to exhibit a localised effect on proximal trunk stability via an enhanced feedforward mechanism at low levels (< 25%) of maximal voluntary contractions (MVC) [6], the concept of discrete trunk muscle training (TMT) has subsequently been deployed to improve physical fitness [7, 8] as well as sport-specific performance. In this regard, earlier approaches defined a muscular cylinder within the trunk formed by a group of muscles, comprising the diaphragm, pelvic floor, abdominals, paraspinals, and gluteals [9–11]. Accordingly, the term “core training” has become widely used to describe exercises that broadly focus on augmenting proximal stability (i.e., trunk stiffness) during limb-loading tasks [6]. Initial research examined the active role of isolated, anatomically deep-lying muscles in spinal stability, as well as the contribution of passive (i.e., non-contractile) elements (ligaments and bone) attached to the spine, pelvis, and hips [9, 10, 12]. However, “core training” does not adequately distinguish between anatomical depth or localization of target muscles [13]. Given the difference in emphasis between these approaches (low-load, muscle-specific proximal stability training vs. global strength and/or muscle endurance training of the whole trunk), the present review has adopted the term TMT, as opposed to “core training,” to describe exercise regimens that focus on trunk muscles (i.e., axial components of the skeleton), irrespective of anatomical orientation and depth.

Trunk muscles provide proximal stability for distal mobility, facilitating transfer of torque and angular momenta between the limbs [12, 14]. Consequently, the trunk has been described as a “powerhouse” due to its capacity to transfer, absorb, and redirect kinetic energy during functional activities [4, 5, 10]. As a kinetic link, connecting upper and lower extremities during whole-body movements, the trunk plays a crucial role in acquisition and execution of sport-specific skills, as well as during sports performance, fitness training, and activities of daily living [4, 14, 15]. Despite the number of studies acknowledging the importance of TMT for sport-specific performance, the available evidence is inconclusive [16, 17]. In golf [18] and handball the significant improvements in drive distance and throwing velocity were 4.8% and 4.9% after a TMT intervention, whereas no significant improvement was observed in swimming (50-m crawl time) [19, 20] or rowing (e.g., 2000-m ergometer time) [17].

During the last decades, original papers and reviews of different TMT strategies have been conducted [3, 12, 14, 15, 21, 22]. However, several of these studies included healthy and active participants of low sport-specific expertise level, rather than competitive athletes [12, 15, 23]. Therefore, these findings cannot be generalized to other populations, particularly highly trained and elite athletes. Furthermore, lack of homogeneity between TMT interventions in terms of weekly training frequency, length of each training session, and number of sets and repetitions [12, 15, 23] could explain the inconclusive findings in the literature [16–20]. In addition, debate as to whether TMT should be applied as an isolated modality or as part of compound, multi-joint training programs (e.g., those including deadlift, squat, bent-over row) is on-going. For deadlift and squat exercises, cross-sectional studies have demonstrated greater trunk muscle activation during the performance of heavy resistance strength exercises compared with isolated trunk muscle exercises [24, 25]. Still, and to the authors’ best knowledge, neither dose–response relation effects of TMT nor longitudinal effects of integrated TMT compared to isolated TMT have been addressed in the literature.

Furthermore, differences in performance level (e.g., elite athletes vs. recreational athletes) and age (e.g., youths vs. adults) have not been included previously within explanatory models to examine their potential moderating effects on TMT outcomes. When applying similar training stimuli in participants of different training expertise and/or fitness status, less experienced individuals achieve larger magnitudes of improvements than more experienced subjects [26]. A potential ceiling effect for TMT to deliver performance-related gains has been reported to have an impact on magnitude of adaptive responses in individuals of higher fitness status [26, 27]; however, this phenomenon has not been formally examined by including athletes of different ages undergoing TMT [28]. Mechanistically, sport-specific benefits of TMT could be attributed to improved locomotor efficiency as a result of reductions in non-sagittal displacement (e.g., trunk rotation and lateral flexion), leading to greater dynamic trunk stability and reduction in energy cost during sport-specific actions, which involve trunk perturbation. As maturation is ongoing in young athletes, unlike their adult counterparts, it is possible that TMT exerts a greater effect on task performance in younger subjects, by dampening proximal perturbation associated with sagittal limb actions, in the presence of continuous and/or high-velocity linear and appendicular growth phases [29]. However, studies comparing the effects of TMT on different age groups are missing in the literature.

Only a few reviews on TMT have been conducted previously [3, 4, 12, 14, 15, 21, 22], which included a variety of cohorts [3, 12, 22], and focused either on TMT for performance development [14, 21], or injury prevention or rehabilitation [4]. To the authors’ knowledge, only one meta-analysis has been conducted that included trained individuals [23]. More specifically, the systematic review and meta-analysis of Prieske et al. [23] reported small effect sizes for the relationship between trunk muscle strength and physical fitness and small-to-moderate effects of TMT on physical fitness. However, Prieske et al. [23] included both healthy (recreationally) trained individuals and competitive athletes. Furthermore, the study did not perform sub-group analyses for moderator variables, such as age, sex, and expertise level, which are likely to modulate TMT effects in competitive athletes [30–33]. Sex-specific differences in anabolic hormones after onset of puberty, age, and maturation (e.g., pre-pubertal, pubertal, and post-pubertal), and training expertise and/or fitness status (e.g., ceiling effect), have resulted in different strength gains [26, 34, 35]. Whether these moderator variables (e.g., age, sex, and expertise level) affect TMT has not been addressed in the literature.

Therefore, the main aim of this systematic review and meta-analysis was to examine the effects of TMT on physical fitness and sport-specific performance in apparently healthy, competitive athletes. In addition, sub-group analyses, which are lacking in the literature, were proposed, to investigate whether participant characteristics such as age, sex, and expertise level were significant moderators of TMT effects, and to examine the effect of training-related programming parameters, such as weekly training frequency, session duration, and chronic training exposure, on TMT outcomes.

## Methods

The present study followed the Preferred Reporting Items for Systematic Reviews and Meta-Analyses Protocols guidelines [36, 37]. The review protocol was not registered, as systematic reviews assessing sport performance are not accepted with PROSPERO.

### Search Strategy

A systematic search was conducted in PubMed, Web of Science, and SPORTDiscus. The computerized search was conducted by an independent researcher using keywords related to TMT, physical fitness, and sport-related performance measurements. Previous reviews and meta-analyses [15, 23] were used to help define our search strategy, which was conducted using the following Boolean operators “AND,” and “OR”: (“core training” OR “core stability” OR “core endurance” OR “core strength” OR “trunk training” OR “trunk stability” OR “trunk endurance” OR “trunk strength”) AND (“athletes” OR “players”) AND (“performance” OR “velocity” OR “speed” OR “height” OR “distance” OR “time”) AND (“training intervention” OR “training period”). In addition, we analyzed relevant review articles published before June 2021 [4, 12, 14, 15, 21, 23], to identify additional studies potentially eligible to be included in this systematic review. In addition, reference lists from all identified articles were screened for publications not identified by the original computerized search. Finally, we asked two independent experts (ML; MB) in the field to provide a list of five key papers within the scope of this review. The two lists were used to ensure that we identified all relevant papers. The search was limited to English and Scandinavian languages. Only original, full-text articles with human participants were included, with no restriction on publication year. Conference abstracts, unpublished studies, pilot studies, or studies not published in peer-review journals were excluded. A secondary search was conducted, approximately 2 weeks before the submission of the paper, to ensure that all recently published papers were identified. Two potential papers were identified [38, 39] and one of these papers was included in the analysis [38].

### Selection Criteria

To be included, studies had to meet the following criteria: Participants: (1) active and apparently healthy competitive athletes, free from injury; (2) aged > 10 and < 40 years; (3) intervention: supplementary TMT (minimum ten sessions over at least 6 weeks) in addition to regular training; (4) reported performance indicator (measure of physical fitness or sport-specific performance); (5) included comparator (passive, active, alternative training). Studies with a two-armed TMT intervention design were excluded, as none of the interventions could serve as a control condition. Furthermore, TMT was defined as a training program incorporating specific exercises (e.g., body mass, slings, medicine balls, fitness balls) with the primary objective of targeting ventral, dorsal, and lateral muscles of the trunk (e.g., abdominal curl, side-bridge, prone bridge). Studies only including whole body resistance exercises (e.g., Olympic lifts, squats, bench press, rowing, and deadlifts) were not included.

**Fig. 1:**
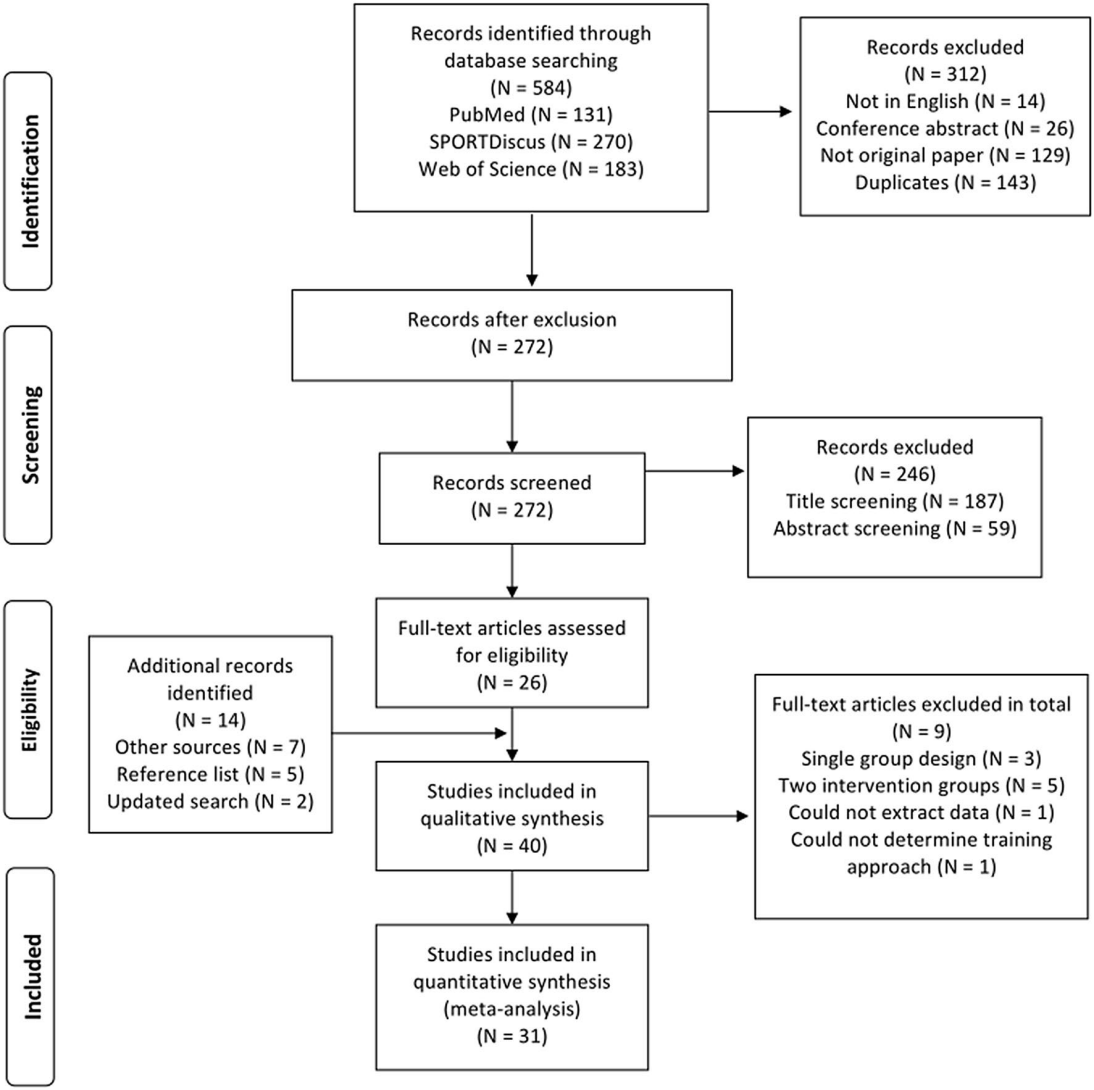
Flowchart illustrating the different phases of the search and selection strategy

The search strategy discovered several articles (see Fig. 1), but many of the identified studies were excluded because: (1) investigators implemented a strength training program where the majority of the exercises did not specifically target trunk muscles; (2) the study design was not a controlled trial; (3) injured athletes or patients were recruited; (4) means and standard deviations were not consistently reported in results (and the authors did not respond to our inquiries); (5) child athletes aged < 10 years or adult athletes aged > 40 years were included; (6) no measures of physical fitness or sport-specific performance were assessed; and (7) participants were not competitive athletes. An overview of the excluded and included studies is presented in Fig. 1.

### Study Quality

Two independent reviewers (AHS and NS) assessed the risk of bias and methodological quality of eligible articles using the Physical Therapy Evidence Database (PEDro) scale [40]. Scores were assigned, based on assessing each study against the eleven criteria used to rate internal and external variability, on a scale from 0 (high risk of bias) to 10 (low risk of bias). A score of 6 or more represents the threshold for studies with low risk of bias [41, 42]. In training intervention studies, it is impossible to blind participants to an exercise program, and the investigators are rarely blinded. Therefore, we removed PEDro scale items 5, 6, and 7, which reduced the maximal score to 7. Based on previous reviews of exercise interventions [41, 42], studies with scores were interpreted as follows: 6–7 “excellent quality”, 5 “good quality, 4 “moderate quality”, and 0–3 “poor quality.” If possible, we aimed to include studies with a score ≥ 6 from the PEDro Scale (i.e., 0–7); however, the score itself was not a criterion for inclusion or exclusion. Points were only awarded if a study clearly met the criteria. If there was disagreement between reviewers (AHS and NS) with regards to the rating, a third assessor (VA) was contacted to achieve a consensus through discussion.

### Data Extraction

A template from previous systematic reviews and meta-analyses conducted by our research group was used to extract data [23]. One author (AHS) extracted the data from the included studies, and a second author (NS) double-checked the extracted data. Disagreements were resolved through personal communication between the two authors (AHS, NS). Each study was coded for the following variables: sport, expertise level, number of participants, age, sex, sport-specific performance measures, physical fitness outcomes, and trunk training endurance (e.g., time to fatigue in prone bridge or side-bridge position). Physical fitness outcomes were divided into the following categories: lower limb muscle power (power output, vertical- and horizontal jumping performance, and acceleration over 0–10 m), linear sprint speed (20–40 m sprint), change-of-direction speed (CODS/agility), local muscle endurance (e.g., numbers of push-ups), and muscle maximal strength (e.g., maximal isometric contraction of the hip and leg, isokinetic torque of hip (flexion and extension), and during a dynamic action (squat and leg press)). Sport-specific performance measures included throwing velocity, drive distance (golf), and race time (rowing and swimming).

In addition, programming parameters, such as weekly training frequency, duration of the intervention, total number of training sessions, and duration per session, were extracted. According to Thiele et al. [43] and Cochrane decision rules [44], multiple outcomes were ranked, based on the most significant outcome for sport-specific performance. Furthermore, if performance outcomes were divided by sex, without reporting a mean of the groups, data were merged to provide one independent outcome for each group [36]. Similarly, if trunk outcomes reported both left and right side (e.g., side-bridge) in a test, results were merged using the same approach.

### Statistical Analyses

To determine the effects of TMT on physical fitness and sport-specific performance, between-subject standardized mean differences (SMDs) were calculated, according to the following equation: SMD = (mean1 – mean2)/*s*_pooled_ [23, 45, 46], with ‘mean1’ defined as the mean pre/post-test value of the intervention group, ‘mean2’ as the mean pre/post-test value of the control group, and ‘*s*_pooled_’ as the pooled standard deviation. In accordance with Hedges and Olkin [47], the SMD was adjusted for sample size using the factor (1 – (3 /4 *N* − 9)), with *N* representing the total sample size. Additionally, adjusted SMD values were calculated as the difference between pre-test SMD to post-test SMD [48]. Finally, a random effects model was applied to weight each included study according to the magnitude of the respective standard error, and to aggregate weighted, mean adjusted SMDs. At least two intervention groups had to be included in order to aggregate SMD values for each proxy of physical fitness [44]. The meta-analysis was conducted using Review Manager 5.3 (The Nordic Cochrane Centre, The Cochrane Collaboration, Copenhagen, Denmark).

Sub-group analyses were computed for subject-related moderator variables (i.e., age, sex, performance level) and training-related programming parameters (i.e., training period, weekly training frequency, total number of training sessions, session duration). More specifically, participants were classified as children (> 10 to ≤ 13 years), adolescents (> 13 to ≤ 18 years), and adults (> 18 years), based on previous classifications [33]. Sex has previously been used as a moderator variable due to potential differences in training-related adaptations to resistance training [30]. Participants’ expertise level (i.e., level of competition: regional, national, international) was included as a moderator variable, on the basis that expertise level has been reported to have an impact on the magnitude of adaptive responses (i.e., ceiling effects if competitive level) [26, 27]. Athletes' expertise level was classified as either elite or non-elite (i.e., recreational and sub-elite) [43]. Elite athletes were defined as athletes competing at a national and/or international level [49].

To analyze training programming parameters in relation to TMT, single-factor analyses were conducted on the following parameters: training period (i.e., ≤ 8 vs. > 8 weeks), number of weekly training sessions conducted (i.e., 2 sessions per week vs. 3 session per week vs. ≥ 4 sessions per week), total number of training sessions (i.e., ≤ 18 vs. > 18 sessions) and session duration (≤ 30 vs. > 30 min) [30, 33].

To improve readability, we consistently reported positive SMD values if a favorable effect of TMT, compared with controls, was indicated. A *p* value of < 0.05 indicated a statistically significant effect. SMD values were classified as trivial (SMD < 0.2), small (0.2 ≤ SMD < 0.5), medium (0.5 ≤ SMD < 0.8), and large (SMD ≥ 0.8) [50].

The level of between-study heterogeneity was assessed using the *I*^2^ statistics [51]. *I*^2^ outcomes of 25, 50, and 75% correspond to low, moderate, and high heterogeneity [52]. Values above 75% were rated as heterogeneous. In addition, a chi-square statistic ($\chi^{2}$) was included to determine whether the results of the analysis were due to chance. In such cases, low *p* values, or high $\chi^{2}$ statistics, relative to degrees of freedom (*df*), would be observed.

## Results

### Study Characteristics

The flow chart illustrates the systematic search process (Fig. 1). The search identified 584 potential papers, of which 31 studies [16–20, 38, 53–77] met the inclusion criteria (Fig. 1). The included studies comprised 693 young and adult athletes (*n* = 369 in intervention group, *n* = 324 in control group) from different sport disciplines (Tables 1, 2 and 3). As expected in this setting, none of the control groups were passive and all continued their normal sport training routines (i.e., active controls). For the sub-group analysis according to age, eight studies included children (*n* = 177; Table 1), eight studies included adolescents (*n* = 158; Table 2), and 14 studies included adults (*n* = 358; Table 3). For sub-group analysis according to sex, six studies included females (*n* = 175), 16 studies included males (*n* = 363), and six studies included both sexes (*n* = 134). In terms of athletes’ expertise level, four studies were categorized as elite (*n* = 106) and 27 studies as sub-elite/recreational (*n* = 587). In addition, eight studies reported 1–5 years of the specific-sport experience [17, 57, 67, 69, 72, 74, 75, 77], six studies reported between five and 10 years [16, 18, 19, 38, 62, 71], and two studies reported over 10 years [53, 60], whereas 15 studies did not report the duration of sport-specific experience of the participants [20, 54–56, 58, 59, 61, 63–66, 68, 70, 73, 76].

**Table 1:** Overview of included studies of children (aged > 10 to ≤ 13 years)

Study	Sport	Number of subjects	Age (years)	Performance level	Sport performance outcome	Trunk tests	Intervention	PEDro score
Bayrakadar and Boz [76]	Soccer	30 males CON = 10 TMT static = 10 TMT dynamic = 10	12–14	Sub-elite/Recreational	30-m sprint, horizontal jump, vertical jump, Agility test (550 test)	Biering-Sorensen test, prone bridge, sit-ups	9 weeks, 2x × week, 30 min, 6 exercises, 2 sets of 20–60-s hold time or 2 sets 10–25 reps	5
Farhan et al. [75]	Soccer	25 males CON = 12 TMT = 13	13 ± 0.5	Sub-elite/Recreational	Agility test (Illinois), Horizontal jump, vertical jump, 20 m sprint	Prone bridge	12 weeks, 5x × week, 20–25 min, 5-s hold, 2–5 reps	7
Fernandez-Fernandez et al. [72]	Tennis	30 males CON = 15 TMT = 15	CON; 13 ± 0.6 TMT; 13 ± 0.5	Sub-elite/Recreational	Serve velocity, Serve accuracy, ROM in shoulder rotation		6 weeks, 3x × weeks, 10–15 min, 2 sets of 20 reps or 3 sets of 20-s hold time, 25 min with upper body strength (2 sets with 20 reps) and 20 min with medicine ball throw exercises	7
Genç and Ciğerci [70]	Soccer	20 males CON = 10 TMT = 10	CON; 13 ± 0.7 TMT; 13 ± 0.6	Sub-elite/Recreational	Acceleration (0–10 m), 30 m -m sprint, Push-ups, Horizontal jum	Prone bridge	8 weeks, 3x × week, 60 min, 6–12 dynamic exercises, 2 sets with 10–35 reps	5
Gencer, [69]	Swimming	24 females CON* = 12 TMT* = 12	CON; 11 ± 1.3 TMT; 11 ± 1.3	Sub-elite/Recreational	50 m -m free style, Horizontal jump, Vertical jump, Push-ups, 30 m -m sprint		8 weeks, 5x × week, 20 min 7 low- intensity dynamic and isometric exercises	5
Hoshikawa et al. [71]	Soccer	28 males CON = 12 TMT = 16	12–13	Sub-elite/Recreational	Jumping (CMJ and SJ), 15 m -m sprint	Hip torque (flexion and extension)	6 months, 4x × week, 3 isometric exercises, 1–2 sets, 30-s hold time	5
Ozmen and Aydogmus [73]	Badminton	20 mixed sex CON = 10 TMT = 10	CON; 11 ± 0.4 TMT; 11 ± 0.3	Sub-elite/Recreational	Agility test (Illinois)	Star Excursion Balance test, endurance test (side bridge)	6 weeks, 2x × week, 20 reps or 20-s hold time	7
Panagoulis et al. [77]	Soccer	28 males CON = 14 TMT = 14	CON; 11 ± 0.6 TMT; 11 ± 0.5	Sub-elite/Recreational	Jumping (CMJ and SJ), Acceleration (0–10 m), 20 m -m sprint, Kicking velocity, 1RM squat		8 weeks, 3x × week, 20 min. Dynamic and isometric plank exercises, 3 sets, 8 reps or 15-s hold time + lower body resistance exercises	7

**Table 2:** Overview of included studies of adolescent (aged > 13 to ≤ 18 years) athletes

Study	Sport	Number of subjects	Age (years)	Performance level	Sport-specific performance	Physical fitness	Trunk tests	Intervention	PEDro score
Afyon [63]	Soccer	30 males CON = 15 TMT = 15	16	Sub-elite/Recreational		CMJ Horizontal jump 20-m sprint	Balance (Flamingo), Prone bride time	12 weeks, 3x × week, 30–35 min. 1–3 sets, 15–35 s duration, isometric and dynamic exercises	4
Aslan et al. [68]	Soccer	29 males CON = 14 TMT = 15	CON; 16 ± 0.8 TMT; 16 ± 0.6	Sub-elite/Recreational		Pro agility, shuttle run test, Horizontal jump Triple-hop distance	Balance error scoring system test	8 weeks, 3x × week, 2 sets, 10–20 reps and 2–3 sets with 25–40 s hold time	7
Clark et al. [56]	Cross-country running	33 mix mixed sex CON *n* = 17 TMT *n* = 16	Males 16 ± 1.3 Females 17 ± 1.3	Sub-elite/Recreational	Race time 5-km course (males) and 4- km course (females)		Isometric force in the hip (abductors, adductors, extensors and flexion)	6 weeks, 3x × weeks, 8 dynamic exercises (1–2 sets of 5–10 reps) and 2 isometric exercises (30 s hold time x × 10 reps)	6
Genc et al. [65]	Handball	10 females CON = 10 TMT = 10	CON; 18 ± 1.9 TMT; 18 ± 1.4	Sub-elite/Recreational		Agility, CMJ, Horizontal jump 10- and 30-m sprint	Prone bride, Biering-Sorensen test, Star Excursion Balance test	8 weeks, 3x × week, 30 min. 6–12 exercises, 2 sets, 10–35 repetitions	5
Ozmen et al. [74]	Handball	20 males CON = 10 TMT = 10	CON; 15 ± 0.5 TMT; 15 ± 0.3	Sub-elite/Recreational	Throwing velocity (standing, 3 steps and jump),	Squat jump	Star Excursion Balance test, Side bridge	6 weeks, 2x × week, 10–20 reps and 20 s hold time	5
Patil et al. [20]	Swimming	60 mix mixed sex CON = 30 TMT = 30	CON males; 15 ± 1.3 CON females; 13 ± 1.5 TMT males; 15 ± 1.3 TMT females; 13 ± 1.5	Sub-elite/Recreational	50-m front crawl, stroke rate, stroke length		Prone bridge	6 weeks, 3x × week, 30–60 min, 6 exercises, isometric exercises with hold time between 15 and 60 s	7
Saeterbakken et al. [16]	Handball	24 females CON *n* = 10 TMT *n* = 14	17 ± 0.3	Sub-elite/Recreational	Throwing velocity (standing)			6 weeks, 2x × week, 60 min. 6 dynamic exercises, 4 sets of 4–6 repetitions	5
Weston et al. [19]	Swimming	20 mix mixed sex CON *n* = 10 TMT *n* = 10	16 ± 1. 0	Elite	50-m swim time		Prone bridge time	12 weeks, 3x × week, 30 min. 4 dynamic and 2 Isometric exercises, 2–4 sets, 30–120 s holds or 10–30 repetitions	5

**Table 3:** Overview of included studies of adult (aged ≤ 18 years) athletes

Study	Sport	Number of subjects	Age (years)	Performance level	Sport-specific performance	Physical fitness	Trunk tests	Trunk training intervention	PEDro score
Afyon [64]	Soccer	36 males CON *n* = 18 TMT *n* = 18	CON; 22 ± 2.6 TMT; 21 ± 2.1	Sub-elite/Recreational		CMJ MVIC leg	Isometric back strength	8 weeks, 2x × week, 15–20 min. 10 exercises, 1–3 sets, 12–20 s duration or 8–20 repetition	3
Butcher et al. [62]	Mix	55 (20 males, 35 females) CON *n* = 14 TMT *n* = 14 TMT + LLS = 14 LSS *n* = 13	CON; 24 ± 4 TMT; 23 ± 3 TMT + LLS; 23 ± 4 LLS; 21 ± 3	Sub-elite/Recreational		Vertical takeoff speed Leg press strength	Modified double straight leg-lowering	TMT: 9 weeks, 3x × week, 3 sets of 5 reps	5
Dağanay et al. [38]	Soccer	24 males CON = 12 TMT = 12	CON; 18 ± 0.8 TMT; 18 ± 0.7	Sub-elite/Recreational		Linear speed (40 m) Agility (*T*-test) Agility (Hexagon test)		8 weeks, 3x × week, 30–35 min, 10 dynamic and static exercises, 2–3 sets with 10–30 reps with an progressive intensity (50–80% of max)	6
Hung et al. [57]	Mix (long-distance runners, football, basketball and rugby	21 males CON *n* = 10 TMT *n* = 11	21 ± 2.0	Sub-elite/Recreational		*V*O_2max_, blood lactate concentration, heart rate, RPE	Balance (3 conditions with eyes open and 3 with eyes closed), time to failure in a sport specific plank test (SEPT)	8 weeks, 2–4x × week, 30 min, dynamic and isometric exercises, 3 sets, 20–25 reps and 3 sets, 30–60 s holds	5
Karpiński et al. [60]	Swimming	16 males CON *n* = 8 TMT *n* = 8	CON; 20 ± 1.9 TMT; 20 ± 1.2	Elite	50-m front crawl	Starting kinematics		6 weeks, 3x × week, 25 min. 4 exercises, 40 s work, 20 s pause x × 4 set.s	7
Kim [59]	Golf	17 females CON *n* = 8 TMT *n* = 9	CON; 22 ± 4.4 TMT; 23 ± 3.7	Elite	Driving distance		Flexibility (flexion and extension), isotonic, and isometric strength lower back	12 weeks, 3x × week, 60 min, 7 dynamic exercises, 3 sets, 12–14 reps. In addition; dead lift and squat (60% of 1RM, 3 sets, 12–14 reps)	4
Kuhn et al. [53]	Handball	20 females CON *n* = 10, TMT *n* = 10	23 ± 4.4	Sub-elite/Recreational	Throwing velocity; standing and jump throw		Isometric strength, time to failure in ventral, dorsal and lateral (plank) positions	6 weeks, 2x × week, 45 min. 4 dynamic and 4 isometric exercises, 2 sets of 45 s holds or duration	7
Manchado et al. [54]	Handball	30 males CON *n* = 15 TMT *n* = 15	19 ± 7.7	Sub-elite/Recreational	Throwing velocity; standing throw, with run-up and jump throw			10 weeks, 3x × week, 10–25 min. 7 dynamic and isometric exercises	7
Mills et al. [61]	Volley- and basketball	30 females CON *n* = 10 TMT 1 *n* = 10 TMT 2 *n* = 10	CON; 19 ± 1.7 TMT1; 20 ± 2.0 TMT2; 19 ± 1.1	Sub-elite/Recreational		Agility (*T*-test) Vertical jump (Sargent`s test)	Balance (Bass stick test)	10 weeks, 4x × week. TMT 1; LMS exercises with isometric and dynamic exercises VTG 2; GMS exercises 12–40 reps, 2–3 sets	6
Sato and Mokha [58]	Running	20 (males and females) CON *n* = 12 TMT *n* = 8	37 ± 9.4	Sub-elite/Recreational	5000-m performance		Star excursion balance test	6 weeks, 4x × week, 5 dynamic exercises, 2–3 sets, 10–15 reps	5
Sharma et al. [49]	Volleyball	40 (20 males and 20 females) CON *n* = 20 TMT *n* = 20	22 ± 1.8	Sub-elite/Recreational		CMJ, squat jump, spike jump and block jump	Static balance standing on a wobble board	9 weeks, 5x × week, 30–40 min. 10–16 dynamic exercise, 4 sets, 10 reps	7
Sung et al. [18]	Golf	60 males CON *n* = 20 TMT *n* = 20	CON; 24 ± 1.0 TMT; 23 ± 0.5	Elite	Drive distance	Peak isokinetic torque of non-dominant arm (wrist, elbow. and shoulder)	Peak isokinetic torque in the trunk (extension and flexion)	8 weeks, 3x × week, 60 min, 5 dynamic exercises (3 sets, 12–15 reps)	7
Taskin [67]	Soccer	40 females CON = 20 TMT = 20	CON; 19 ± 0.8 TMT; 19 ± 1.2	Sub-elite/Recreational		30-m sprint aAcceleration (0–10 m) Squat jump Horizontal jump		8 weeks, 3x × week, 2 sets, 10–15 reps or 25–40- sec hold time	7
Tse et al. [17]	Rowing	34 CON *n* = 20 TMT *n* = 14	CON; 20 ± 1.0 TMT; 21 ± 1.0	Sub-elite/Recreational	2,000-m row performance including time, blood lactate concentration, and heart rate	40-m sprint, shuttle run and lower-body power	Overhead medicine ball throw, trunk endurance (flexion, extension, and later flexion)	8 weeks, 2x × week, 30–40 min. Trunk stability and endurance exercises In addition, both CON and TMT conducted a circuit training program (2 sets, 12–15 reps, 50% of max)	3
Vignesh-waren [60]	Soccer	20 males CON = 10 TMT = 10	22–24	Sub-elite/Recreational		50-m sprint		6 weeks, 5x × week, 30 min, in addition to two plyometric lower-body exercises	6

Twenty-four of the 31 included studies reported greater numbers of training sessions for the TMT group than the active controls [17–19, 38, 53–58, 60, 62–67, 69–74, 76]. Three studies reported similar weekly training sessions (i.e., training time for TMT replaced sport-specific training) [16, 75, 77], and four studies did not report the weekly number of training sessions [20, 59, 61, 68]. The mean duration of TMT was 8.6 weeks (± 3.5; range 6–24) and mean weekly frequency was 3.1 sessions (± 0.8; range 2–5); mean total number of TMT sessions was 27.9 (± 17.0; range 12–96). Nine studies reported a session duration between 10 and 30 min, nine between 30 and 60 min, and two studies over 60 min. Eleven studies did not report session duration.

In terms of quality assessment using the PEDro scale (Tables 1, 2 and 3), the median quality score was 5 points (95% confidence interval (CI) 4.8–5.5), which can be classified as good methodological quality with low risk of bias. Twenty-three of the studies reached the pre-determined cut-off score of ≥ 5 points. In terms of quality, 14 studies were scored as “excellent” (≥ 6 points), nine studies as “good” (5 points), five as “moderate” (4 points), and two as “poor” (≤ 3 points).

### Main Analyses

#### Trunk and Local Muscle Endurance

**Fig. 2:**
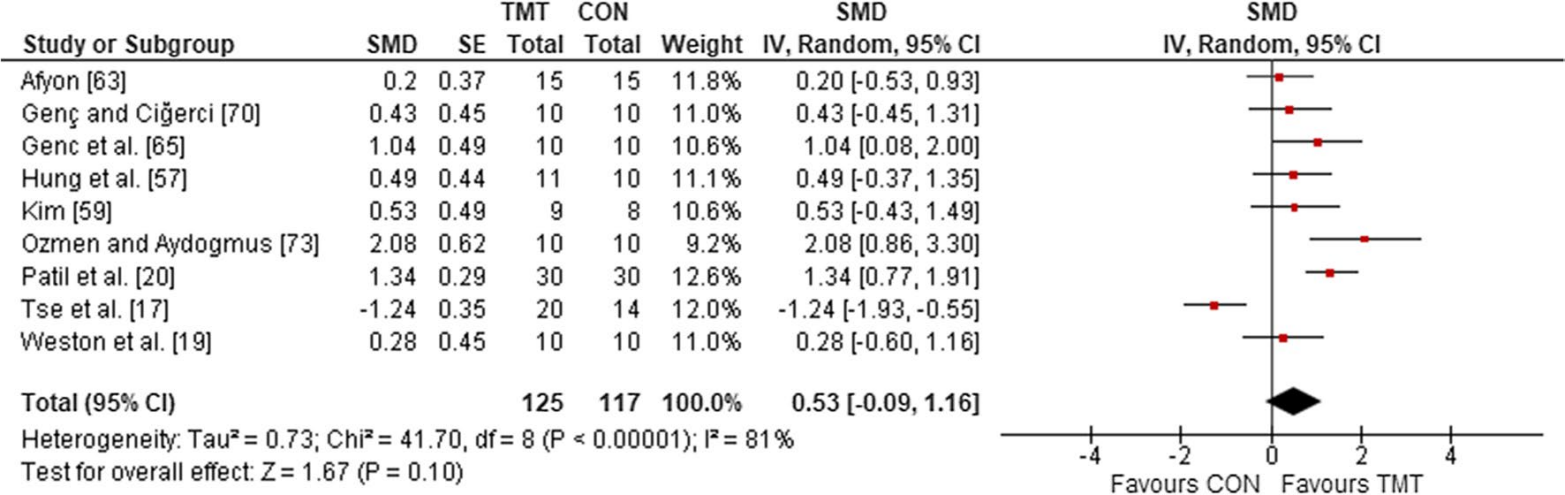
The effects of TMT on trunk muscle endurance compared to regular sport training. *TMT* trunk muscle training, *CON* control group, *CI* confidence interval, *df* degrees of freedom, *IV* inverse variance, *Random* random-effects model, *SE* standard error, *SMD* standardized mean difference

Nine studies were included in the analyses to determine the effects of TMT on trunk muscle endurance, compared to regular sport training. Irrespective of age, sex, and expertise level, the weighted mean SMD of 0.53 (*p* = 0.10; *I*^2^ = 81%, $\chi^{2}$ = 41.70, *df* = 8) indicated a moderate but non-significant effect in favor of TMT (Fig. 2).

**Fig. 3:**
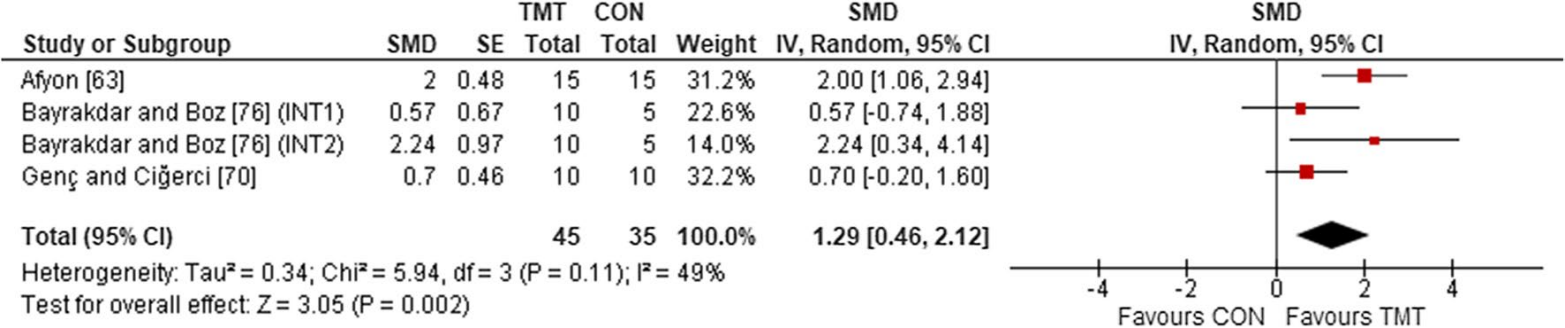
The effects of TMT on local muscular endurance compared to regular sport training. *TMT* trunk muscle training, *CON* control group, *CI* confidence interval, *df* degrees of freedom, *IV* inverse variance, *Random* random effects model, *SE* standard error, *SMD* standardized mean difference, *INT* intervention

Four studies, comprising five intervention groups, were included in the analyses to determine the effects of TMT on local muscular endurance, compared to regular sport training. Irrespective of age, sex, and expertise level, the weighted mean SMD of 1.29 (*p* = 0.002; *I*^2^ = 49%, $\chi^{2}$ = 5.94, *df* = 3) indicated a large effect in favor of TMT (Fig. 3).

#### Strength and Power

**Fig. 4:**
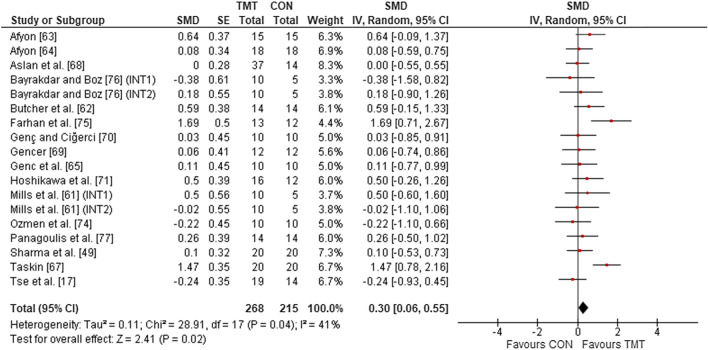
The effects of TMT on lower limb muscle power compared to regular sport training. *TMT* trunk muscle training, *CON* control group, *CI* confidence interval, *df* degrees of freedom, *IV* inverse variance, *Random* random effects model, *SE* standard error, *SMD* standardized mean difference, *INT* intervention

Sixteen studies, with 18 TMT intervention groups, were included in the analyses to determine the effects of TMT on lower limb muscle power, compared to regular sport training. Irrespective of age, sex, and expertise level, the weighted mean SMD of 0.30 (*p* = 0.02; *I*^2^ = 41%, $\chi^{2}$ = 28.91, *df* = 17) indicated a small effect in favor of TMT (Fig. 4).

**Fig. 5:**
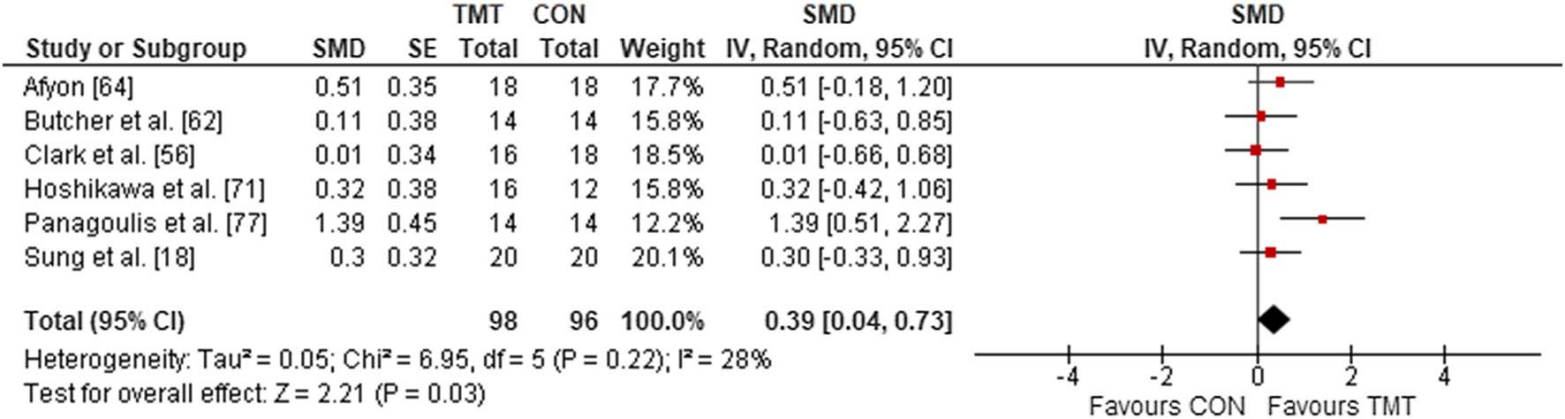
The effects of TMT on maximal muscle strength compared to regular sport training. *TMT* trunk muscle training, *CON* control group, *CI* confidence interval, *df* degrees of freedom, *IV* inverse variance, *Random* random effects model, *SE* standard error, *SMD* standardized mean difference, *INT* intervention

Six studies were included in the meta-analysis to determine the effects of TMT on maximal muscle strength, compared to regular sport training. The overall effects showed a weighted mean SMD of 0.39 (*p* = 0.03; *I*^2^ = 28%, $\chi^{2}$ = 6.95, *df* = 5), which is indicative of a small TMT effect (Fig. 5).

#### Linear Sprint Speed and CODS/Agility

**Fig. 6:**
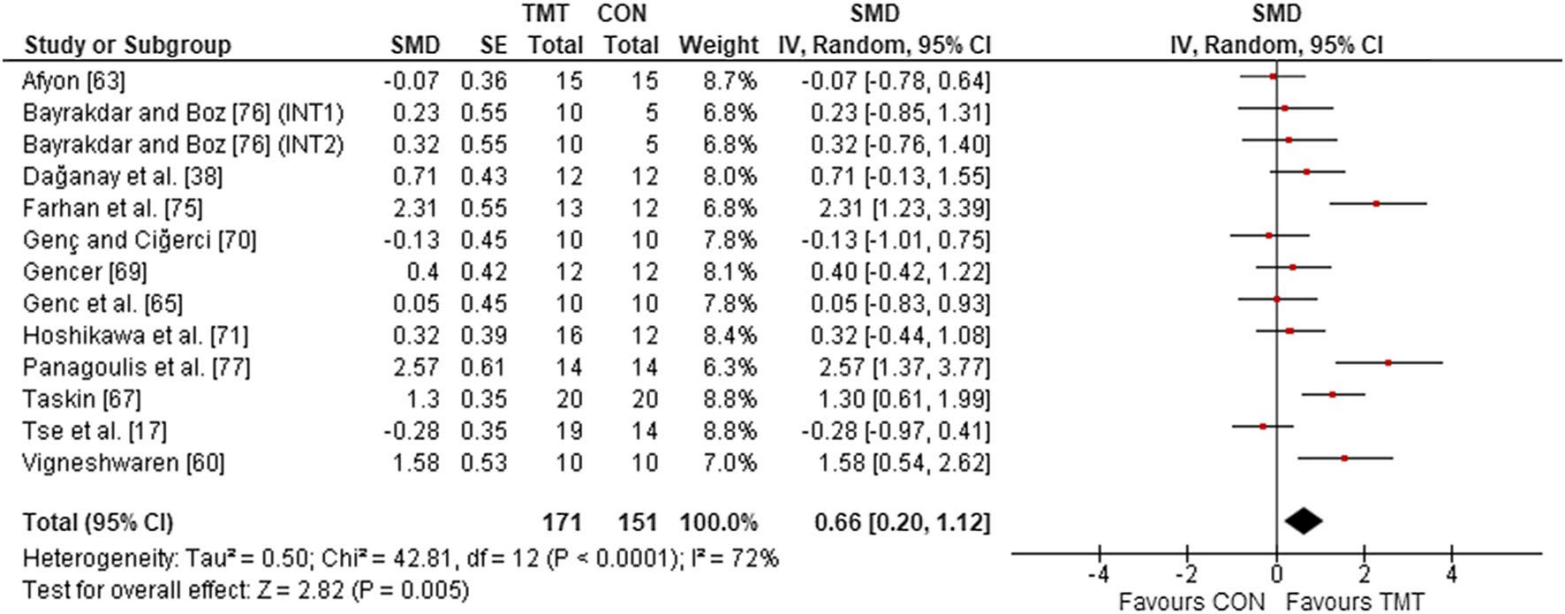
The effects of TMT on linear sprint speed performance compared to regular sport training. *TMT* trunk muscle training, *CON* control group, *CI* confidence interval, *df* degrees of freedom, *IV* inverse variance, *Random* random effects model, *SE* standard error, *SMD* standardized mean difference, *INT* intervention

Twelve studies, with 13 intervention groups, were included in the analyses to determine the effects of TMT on linear sprint speed, compared to regular sport training. The overall effects showed a weighted mean SMD of 0.66 (*p* = 0.005; *I*^2^ = 72%, $\chi^{2}$ = 42.81, *df* = 12), which indicates a moderate TMT effect (Fig. 6).

**Fig. 7:**
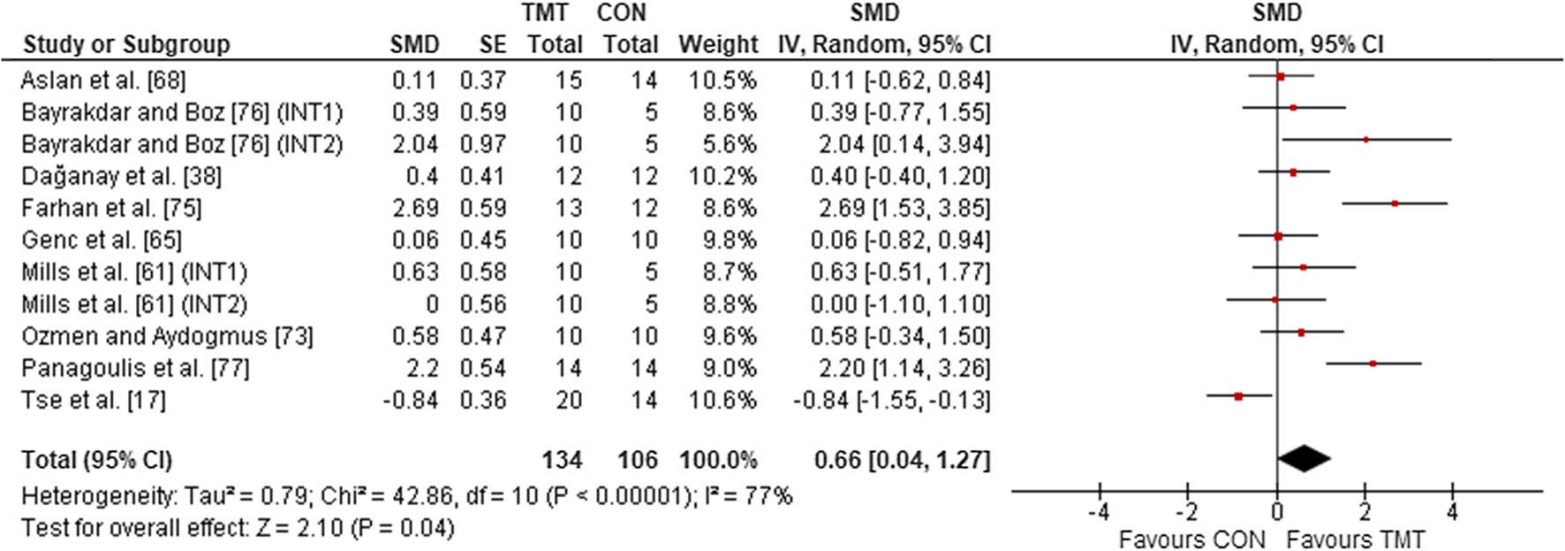
The effects of trunk on CODS/agility performance compared to regular sport training. *TMT* trunk muscle training, *CON* control group, *CI* confidence interval, *df* degrees of freedom, *IV* inverse variance, *Random* random effects model, *SE* standard error, *SMD* standardized mean difference, *INT* intervention

Nine studies, with 11 TMT intervention groups, were included in the analysis to determine the effects of TMT on CODS/agility performance, compared to regular sport training. The overall effects showed a weighted mean SMD of 0.66 (*p* = 0.04; *I*^2^ = 77%, $\chi^{2}$ = 42.86, *df* = 10), which is indicative of a moderate TMT effect (Fig. 7).

#### Sport-Specific Performance

**Fig. 8:**
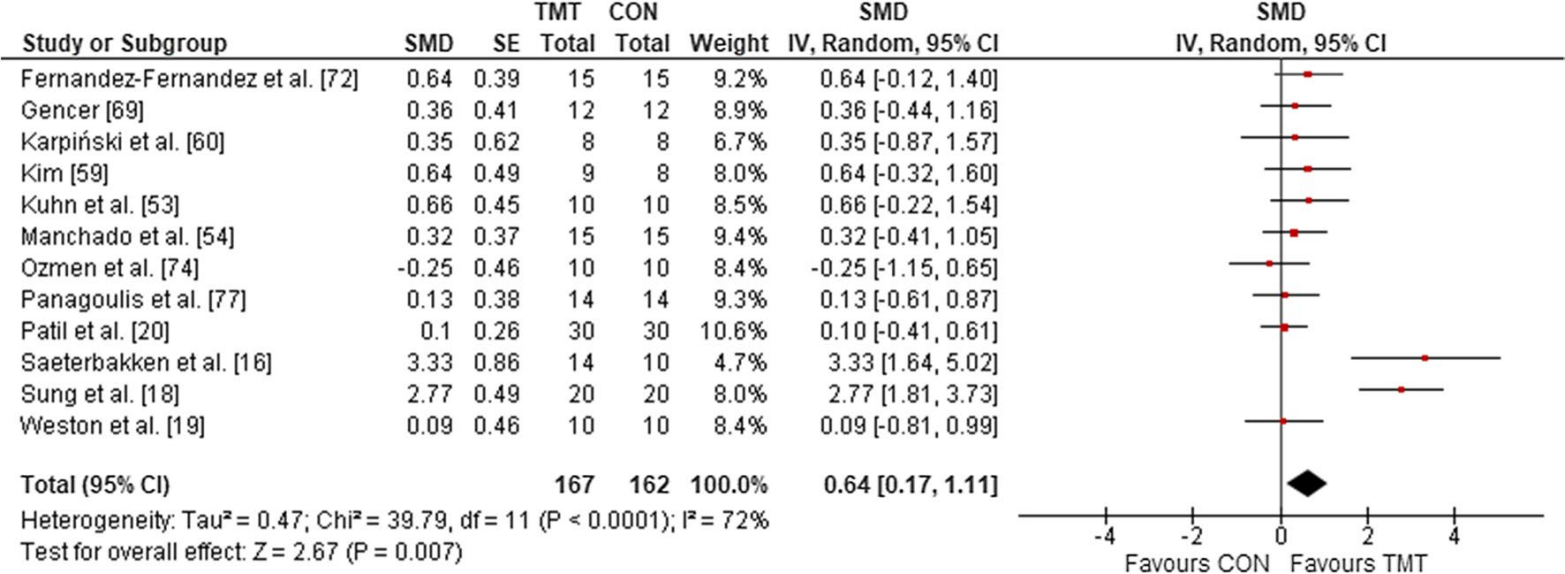
The effects TMT on sport-specific performance compared with regular sport training. *TMT* trunk muscle training, *CON* control group, *CI* confidence interval, *df* degrees of freedom, *IV* inverse variance, *Random* random effects model, *SE* standard error, *SMD* standardized mean difference, *INT* intervention

Twelve studies were included in the meta-analysis that determined the effects of TMT on sport-specific performance, compared with regular sport training only. The analysis demonstrated a weighted mean SMD of 0.64 (*p* = 0.007, *I*^2^ = 72%, $\chi^{2}$ = 39.79, *df* = 11), which indicates a moderate TMT effect (Fig. 8).

### Participant-Related Moderating Variables

An overview of the subject-related moderator variables on TMT effects is displayed in Table 4. Univariate sub-group analysis indicated that age significantly modulated TMT effects, but only for CODS/agility (*p* = 0.04). More specifically, TMT effects on CODS/agility were significant and large-sized in children (SMD 1.53, *p* = 0.002) but not in adolescents and adults (− 0.01 ≤ SMD ≤ 0.09, *p* > 0.05).

**Table 4:** Overall effects of trunk muscle training on measures of physical fitness as well as subgroup-specific effects for subject-related moderator variables

	Trunk muscle endurance			Muscle strength			Muscular endurance			Muscle power/jump			Linear speed			CODS/agility			Sport-specific performance		
	SMD	S (I)	*N*	SMD	S (I)	*N*	SMD	S (I)	*N*	SMD	S (I)	*N*	SMD	S (I)	*N*	SMD	S (I)	*N*	SMD	S (I)	*N*
All	0.53	9 (9)	125	**0.39**	6 (6)	98	**1.29**	3 (4)	45	**0.30**	16 (18)	268	**0.66**	12 (13)	171	**0.66**	9 (11)	134	**0.64**	12 (12)	167
*Sex*		*P* = 0.69			*P* = 0.13			NA			*P* = 0.77			*P* = 0.89			*P* = 0.21			*P* = 0.15	
Females	**0.79**	2 (2)	19	–			–			0.47	4 (5)	62	0.62	3 (3)	42	0.20	2 (3)	30	**1.01**	4 (4)	45
Males	0.32	5 (5)	66	**0.57**	4 (4)	68	**1.29**	3 (4)	45	0.21	10 (11)	172	**0.69**	9 (10)	129	**0.85**	7 (8)	104	0.65	6 (6)	82
Mixed	0.87	2 (2)	40	0.05	2 (2)	30	**–**			0.30	2 (2)	34	–			–			0.10	2 (2)	40
*Age*		*P* = 0.30			*P* = 0.43			*P* = 0.08			*P* = 0.68			*P* = 0.12			***P* = 0.02**			*P* = 0.52	
Children	oEG			0.83	2 (2)	30	**0.90**	2 (3)	30	0.35	6 (7)	85	**0.81**	6 (7)	85	**1.53**	4 (5)	57	0.37	3 (3)	41
Adolescents	**0.93**	5 (5)	75	0.01	1 (1)	16	**2.00**	1 (1)	15	0.14	4 (4)	72	–0.02	2 (2)	25	0.09	2 (2)	25	0.55	4 (4)	64
Adults	− 0.10	3 (3)	40	0.32	3 (3)	52	**–**			0.36	6 (7)	111	0.79	4 (4)	61	–0.01	3 (4)	52	**0.95**	5 (5)	62
*Training status*		*P* = 0.72			*P* = 0.75			*P* = NA			*P* = NA			*P* = NA			*P* = NA			*P* = 0.42	
Elite	0.39	2 (2)	19	oEG			–			–			–			–			0.97	4 (4)	47
Subelite/recreational	0.58	7 (7)	106	0.42	5 (5)	78	**1.29**	3 (4)	45	**0.30**	15 (17)	268	**0.66**	12 (13)	171	**0.66**	9 (11)	134	**0.43**	8 (8)	120

*N* total number of participants in the included experimental groups, *NA* not applicable, *oEG* only one experimental group, *S(I)* number of included studies (number of included experimental groups), *SMD* weighted mean standardised mean difference

bold values indicate significant effects

### Training-Related Programming Parameters

Table 5 displays the effects of training-related programming parameters for TMT-effects on performance-related outcomes. Univariate sub-group analyses revealed that the total number of training sessions significantly moderated TMT effects on lower limb muscle power and linear sprint speed (*p* < 0.05). Significant and small-to-large effects of TMT were found for lower limb muscle power (SMD = 0.45, *p* = 0.003) and linear sprint speed (SMD = 0.84, *p* = 0.002) for > 18 TMT sessions. Furthermore, session duration significantly moderated TMT effects on linear sprint speed, CODS/agility, and sport-specific performance (*p* ≤ 0.05). While TMT sessions of ≤ 30 min duration were found to significantly improve linear sprint speed and CODS/agility (1.66 ≤ SMD ≤ 2.42, *p* ≤ 0.002), sessions lasting > 30 min significantly enhanced sport-specific performance (SMD = 1.22, *p* = 0.008).

**Table 5:** Overall effects of trunk muscle training on measures of physical fitness as well as subgroup-specific effects for training-related moderator variables

	Trunk muscle endurance			Muscle strength			Muscular endurance			Muscle power/jump			Linear speed			CODS/agility			Sport-specific performance		
	SMD	S (I)	*N*	SMD	S (I)	*N*	SMD	S (I)	*N*	SMD	S (I)	*N*	SMD	S (I)	*N*	SMD	S (I)	*N*	SMD	S (I)	*N*
All	0.53	9 (9)	125	**0.39**	6 (6)	98	**1.29**	3 (4)	45	**0.30**	16 (18)	268	**0.66**	12 (13)	171	**0.66**	9 (11)	134	**0.64**	12 (12)	167
*Training period*		*P* = 0.53			*P* = 0.46			*P* = 0.21			*P* = 0.29			*P* = 0.78			*P* = 0.27			*P* = 0.28	
≤ 8 weeks	0.65	6 (6)	91	0.49	4 (4)	68	oEG			0.19	9 (9)	150	**0.72**	8 (8)	107	0.37	6 (6)	81	**0.77**	9 (9)	133
> 8 weeks	0.31	3 (3)	34	0.21	2 (2)	30	**1.57**	2 (3)	35	**0.45**	7 (9)	118	0.58	4 (5)	64	**1.08**	3 (5)	53	0.34	3 (3)	34
*Training frequency*		*P* = 0.87			*P* = 0.93			*P* = 0.94			*P* = 0.70			*P* = 0.07			*P* = 0.73			*P* = 0.72	
2x/week	0.38	2 (2)	30	oEG			1.26	1 (2)	20	0.19	5 (6)	87	– 0.03	2 (3)	39	0.34	3 (4)	50	1.09	3 (3)	34
3x/week	**0.66**	7 (7)	95	0.40	4 (4)	64	**1.34**	2 (2)	25	**0.42**	7 (7)	113	**0.82**	8 (8)	110	0.64	4 (4)	51	0.60	8 (8)	121
≥ 4x/week	–			oEG			–			0.21	4 (5)	68	0.95	2 (2)	22	1.10	2 (3)	33	oEG		
*Training sessions (#)*		*P* = 0.82			*P* = 0.53			*P* = 0.94			***P* = 0.02**			***P* = 0.02**			*P* = 0.46			*P* = 0.82	
≤ 18	**0.69**	3 (3)	60	0.25	2 (2)	34	1.26	1 (2)	20	– 0.10	4 (5)	67	– 0.03	2 (3)	39	0.34	3 (4)	50	0.58	6 (6)	87
> 18	0.46	6 (6)	65	0.48	4 (4)	64	**1.34**	2 (2)	25	**0.45**	12 (13)	201	**0.84**	10 (10)	132	**0.82**	6 (7)	84	0.69	6 (6)	80
*Session duration*		*P* = 0.97			*P* = 0.26			NA			*P* = 0.40			***P* = 0.004**			***P* < 0.001**			***P* = 0.05**	
≤ 30 min	0.39	2 (2)	21	**0.90**	2 (2)	32	–			0.46	4 (4)	57	**1.66**	4 (4)	49	**2.42**	2 (2)	27	0.24	5 (5)	59
> 30 min	0.37	5 (5)	84	oEG			oEG			0.14	4 (4)	64	0.05	4 (4)	56	– 0.15	3 (3)	42	**1.22**	6 (6)	98

*N* total number of participants in the included experimental groups, *NA* not applicable, *oEG* only one experimental group, *S(I)* number of included studies (number of included experimental groups), *SMD* weighted mean standardised mean difference

Bold values indicate significant effects

## Discussion

The aim of this meta-analysis was to examine the effects of TMT on physical fitness and sport-specific performance in apparently healthy competitive young and adult athletes. The analyses showed small-to-moderate-sized effects on measures of physical fitness (e.g., trunk muscle strength) and sport-specific performance (e.g., swimming time) in favor of TMT compared to active controls. Significant between-group differences in the above-mentioned outcomes were observed for age but not for the other two subject-related moderator variables examined (sex and expertise level). In regard to training-related programming parameters, a significant difference according to total number of training sessions was found for TMT effects on muscular power/jump performance and linear speed, and for session duration on linear speed and CODS/agility, but not for intervention duration or weekly training frequency.

Despite the high number of studies included in this meta-analysis, the quality of the studies was acceptable. In fact, 24 of the 31 studies included (Tables 1, 2 and 3) achieved the PEDro cut-off score of > 5 points (“good quality”), with 14 studies achieving a quality score of “excellent” (≥ 6 points), compared with only two studies reported in the previous meta-analysis of Prieske and colleagues [23]. Despite the improvement in methodological quality, and greater number of studies included in this meta-analysis, heterogeneity in training content and programming parameters (i.e., frequency, duration, and length of the intervention), differences in expertise level (recreational/sub-elite vs. elite), sport-performance outcomes, and number of participants recruited, need to be considered when interpreting the present findings. For example, in 24 of the included studies [17–19, 38, 53–58, 60, 62–67, 69–74, 76], weekly TMT sessions were performed in addition to regular training. Potentially, this un-matched supplemental training volume could provide an additional stimulus to induce neurological and morphological adaptations, favoring the TMT groups [26, 27, 78, 79].

### Main Analyses

To the authors’ knowledge, this is the first meta-analysis on TMT that included competitive athletes aged 10–40 years. In analyses of sport-specific outcomes, twelve of the included studies demonstrated moderate effects in favor of TMT (mean SMD of 0.64), which supports findings elsewhere of greater maximal power and more efficient use of the muscles of the lower limbs, shoulders, and arms, following TMT [4, 5, 12]. In comparison, previous meta-analyses have reported small-to-moderate effects [23] and moderate-to-large effects [22] in favor of TMT on muscle power, athletic performance, and functional performance. It is important to consider that Granacher and colleagues [22] included sedentary old adults (≥ 60 years) in their analysis, whereas Prieske et al. [23] included healthy trained individuals (aged 16–44 years). In contrast, this study aggregated data from apparently healthy competitive athletes aged 10–40 years. Furthermore, classification of outcomes, which differed between the present study and Prieske et al. [23], may explain the different effects reported. For example, Prieske et al. [23] classified running/swimming/rowing time trials as sport-specific performances, and high-velocity tasks (i.e., throwing) as muscular power outcomes [23]. In the present study, all these outcomes were merged and categorized as sport-specific outcomes (*I*^2^ = 72%). As reported in the *Results*, assessment of heterogeneity supported the feasibility of this approach. In addition, differences in the number of studies in each outcome category may also explain the discrepancy in findings. For example, Prieske et al. [23] included eight and six studies, in the categories of sport-specific performance and muscular power, respectively, while the present study included 12 and 17 studies, in the categories of sport-specific performance and lower limb muscle power, respectively.

### Sub-Group Analyses of the Subject-Related Moderator Variables

The present study revealed small-to-moderate effects (mean SMD of 0.30–0.70) of TMT compared to regular sport training on CODS/agility, linear sprint speed, and lower limb muscle power/jump performance. Our findings demonstrated similar trends to those in the previously published meta-analysis of Prieske et al. [23], which reported a mean SMD of 0.71 for muscle power in favor of TMT. However, in contrast to Prieske et al. [23], the present study included additional sub-group analyses according to age (children, adolescents, adults), expertise level (elite, sub-elite/recreational), and sex (males, females, or males and females), and therefore extends the findings reported by Prieske et al. [23].

Our results demonstrated between-group differences in the computed sub-group analyses for age, but not expertise level and sex. Children, in contrast to adolescents and adults, demonstrated a significant and large TMT-related effect (SMD of 1.53) of TMT on CODS/agility. Furthermore, trivial-to-small TMT effects (SMD of 0.14–0.36) were observed for lower limb power (including jump performance) across the age categories. Interestingly and irrespective of the non-significant sub-group differences, there were also large TMT effects (mean SMD = 0.81) on linear sprint speed in children, which could be interpreted as suggesting that CODS/agility is highly influenced by linear running speed, and therefore is not an independent outcome category, at least in younger (child) athletes. This proposal is supported by evidence of a moderate association between CODS, linear sprint speed, and an indicator of leg muscle power (jump height) [80], although this was not examined in a youth athlete population. Interestingly, present findings also showed adults demonstrated large TMT effects on linear sprint speed (mean SMD of 0.83), despite the trivial and small TMT effects on CODS/agility and lower limb power. It could be speculated that TMT may improve linear sprint speed by limiting excessive lateral and horizontal trunk displacements; however, in the absence of a concomitant effect on CODS/agility in adults, the present findings suggest that TMT, at least as applied in studies here, did not result in performance gains during tasks involving deceleration and reacceleration, as imposed by COD maneuvers.

Previous meta-analyses have demonstrated greater strength adaptations with older (> 15 years) compared to younger participants (< 15 years) [30], in addition to greater strength gains in pubertal and post-pubertal than pre-pubertal subjects [34]. Different strength-training modalites (e.g., plyometric training, heavy resistance training, power training) have frequently been used to improve CODS/agility and linear sprint speed [80–82], and the results of the present findings are somewhat surprising. Nevertheless, when applying similar training stimuli in participants of different training expertise and/or fitness status, less experienced individuals achieve larger magnitudes of improvements than more experienced subjects [26]. The presence of a ceiling effect may explain the greater TMT effects on CODS/agility in children compared with adolescents or adults.

Unlike the findings of increased CODS and agility for TMT in children, there were no significant, age-related differences of TMT effects on sport-specific performance, linear sprint speed, and lower limb muscle power. Hence, for lower limb muscle power and linear sprint speed, similar non-significant effects (small and large, respectively) were observed between adults and children. For sport-specific performance, adults demonstrated large effects, whereas children demonstrated small effects despite no significant differences. Furthermore, and despite non-significant sub-group effects of performance level, large effects were observed in elite athletes compared to small effects in sub-elite (Table 4). Although only four studies were conducted in athletes at an elite level, underlining the requirement for further research into TMT effects on performance in this population, this finding was somewhat surprising. It is possible to interpret these findings as indicating a role for proximal strength training in enhancing aspects of agility in very young athletes. For example, greater trunk stability after TMT could improve performance during COD maneuvers, as a result of TMT enhancing trunk muscle stiffness, particularly in the abdominal obliques and paraspinals, during locomotor tasks. In very young athletes, unlike their adult counterparts, maturation is ongoing, and therefore it is possible that TMT exerts a greater effect on task performance by dampening proximal perturbation associated with sagittal limb actions, due to the presence of continuous and/or high-velocity linear and appendicular growth phases [29]. Previous studies have demonstrated a proximal to distal movement pattern in sport-specific actions (e.g., gymnastic, throwing, tennis serve), in which trunk muscles are important in the transfer of forces [83–86]. Furthermore, evidence that proximal deficits (“core dysfunction”) significantly influence lower limb function [87], and prospectively are strongly associated with increased risk of lower limb injury [88, 89], indirectly supports the finding of a favorable effect of TMT on athletes’ performance at elite level. Presumably, favorable effects of TMT on athletes’ performance may be linked to more effective force transfer and optimized movement strategies [83–86]. Furthermore, the trunk muscles are used to generate rotational torques around the spine [90–92]. For example, greater gluteus and trapezius muscle activation resulted in increased ankle (26%) [93] and rotator cuff (23–24%) [94, 95] activation, respectively. Potentially, greater trunk muscle strength, and/or stability, may optimize transfer, control, and production of force and kinetic energy during sport-specific movements.

Sub-group analyses according to sex revealed greater, but non-significant effects in females than males for sport-specific performance outcomes (large vs. moderate); however, effects were smaller for CODS/agility (small vs. large) and similar for lower limb muscle power (small) and linear sprint speed (moderate). The cause of the divergence between the sport-specific performance and CODS/agility outcomes between sexes is unclear and may be related to factors such as training volume, training intensity, or expertise level [80–82]. Prieske et al. [23] did not include sex as a moderator in their meta-analysis. Therefore, the present findings are difficult to compare with the latter study. Previously, differences in adaptations to strength training between sexes [30, 34, 96] have been attributed to sex-specific differences in systemic anabolic hormones from the onset of puberty [35]. Furthermore, as a training modality, TMT is not homogeneous, and typically involves different approaches to trunk exercise programming. Despite these differences in approach, adaptation to TMT appears to involve similar morphological and neurological pathways to those elicited by strength training targeting the upper and lower limbs [78, 81].

For maximal muscle strength and trunk muscle endurance outcomes, the lack of studies including measurements of adaptations in trunk muscles makes the comparison between sex, age, and expertise level incomplete (Table 4). Still, time to fatigue in an isometric position (e.g., side-bridge position, prone bridge) was the most frequently used measure of trunk muscle endurance. Although non-significant, females demonstrated greater TMT effects than males in these tests (moderate vs. small effects), adolescents greater than adults (large vs. negative trivial effects), and sub-elite/recreational greater than elite (moderate vs. small effects). Of note, Prieske and colleagues [23] reported a small-sized association between trunk muscle strength and physical performance, in addition to a small-sized correlation (*r* = 0.16, *r*^2^ = 2.6%) between trunk muscle strength and sport-specific performance. The diversity between TMT effects on trunk muscle endurance and physical fitness found in the present analysis may indicate that improved trunk muscle endurance may not necessarily lead to greater sport-related performance. For maximal muscle strength, children displayed large effects, whereas adolescents and adults displayed trivial and small TMT effects. Of note, several of the maximal muscle strength tests included and analyzed in the present paper did not adequately mimic the movement pattern or muscle action generated in sports-related locomotor actions [97].

### Sub-Group Analyses for Training-Related Programming Parameters

It is generally accepted that a dose–response relationship exists between strength training volume and physiological adaptations [27, 98]. However, TMT programs are not necessarily designed to improve cross-sectional area, force output or torque of the trunk, but may also be prescribed to enhance the ability to stabilize the lumbo-pelvic hip complex [4, 5, 14]. For example, during a golf swing, the function of the trunk is not to make the lumbo-pelvic hip complex rigid and stiff via maximal trunk muscle co-activation, but rather to provide fine control and positional alignment of the trunk over the pelvis during motion, to optimize the production, transfer, and control of force. In accordance with this objective, several of the TMT programs reviewed in this study were designed to improve trunk stability, or a combination of strength, stability, and/or endurance. This may in part explain results from sub-group analyses of training-related programming parameters, which did not necessarily support a dose–response relationship for TMT as typically observed for traditional strength-training programs. For example, non-significantly greater effects in favor of shorter TMT periods (≤ 8 weeks) were found for sport-specific performance (moderate vs. small) and trunk muscle endurance (moderate vs. small). Given that the effects of block-periodization of training are well documented [99, 100], with shorter periods at higher training frequencies demonstrated to provide a greater stimulus to adaptation and improvements in performance-related parameters, present findings for categories directly related to sport-specific performance could be partly attributed to lack of evidence of periodization in TMT programming. Of note, similar effects were found for linear sprint speed (moderate) and trunk muscle strength (small); however, there were non-significantly smaller effects on CODS/agility (small vs. large) following shorter (≤ 8 weeks) compared with longer training periods (> 8 weeks). Apparently, the number of sessions conducted was of higher impact than the overall duration of the intervention, with significantly greater effects for more than 18 sessions on linear sprint speed (large vs. trivial) and lower limb muscle power (small vs. negative trivial). Interestingly, for sport-specific performance, similar but moderate effects were found for both less and more than 18 training sessions. Since TMT was performed in addition to, or as a substitute for, regular training sessions it is surprising that fewer (≤ 18) sessions revealed similar effects to those observed for a higher number of TMT sessions (> 18). This could be due to a potential ceiling effect, whereby the effect of TMT on performance-related gains demonstrated saturation or, alternatively, to differences in athletes’ training experience and history, and a wide range of age and/or expertise level [27, 98, 101] included in the studies.

However, there were non-significantly larger effects of TMT at a frequency of two sessions per week compared with moderate effects for three weekly sessions of TMT on sport-specific performance outcomes. Most importantly, effects sizes for sessions with more than 30 min duration were significantly larger compared with TMT effects for shorter (≤ 30 min) sessions (i.e., large vs. small). Although not consistently statistically significant, longer sessions (> 30 min) twice per week appear to be more beneficial for gains in sport-specific performance than shorter sessions at a higher weekly frequency. These findings are difficult to compare directly with what has previously been reported. For example, Prieske and colleagues [23] did not include training-related programming parameters in their analyses. Chaabene et al. [33] did report large effects of strength training programs on CODS, whereas moderate effects were reported for three sessions per week. Elsewhere, in a review of studies that only included females, Moran et al. [30] showed larger effects of strength training on strength performance for fewer training sessions (≤ 16), shorter training periods (≤ 8 weeks), and at lower weekly training frequencies (≤ 2 sessions). It is possible to speculate from this and the present evidence that prescribing TMT at lower frequencies may allow more time for recovery and potentially enhance adaptation responses to the applied TMT training stimuli. Furthermore, periodizing TMT in order to enhance the potential for performance-related gains, according to adaptations demonstrated for other training modalities, is worthy of further investigation.

This study aimed to examine the effects of TMT on physical fitness and sport-specific performance in healthy competitive athletes, and included additional analyses of potential training- and subject-related mediator variables. The present findings support the importance of TMT in improving both sport-specific athlete performance and important indices of physical fitness, specifically muscular strength, muscle power, CODS/agility, and linear sprint speed. Potentially, increased trunk muscle strength and/or enhanced trunk stability as a result of TMT exposure, may be speculated to reduce unwanted trunk displacement (e.g., trunk lateral flexion or rotation) during sport-specific actions, thereby optimizing the efficiency of force transfer between limbs (e.g., from leg to leg, leg to arm, or arm to arm) and across the trunk. Furthermore, of the subject-related moderators examined (sex, age, and performance level), only age significantly modulated a positive TMT effect on CODS/agility. An explanation for this finding could be a potential ceiling effect for TMT, whereby greater longitudinal exposure, and higher volumes of sport training in older elite athletes reduces the potential for TMT to exert a large, independent effect. It is also possible that as studies examining elite athletes are limited, the present investigation was unable to detect the effect of TMT due to heterogeneity within studies and limited numbers of participating subjects. These findings may be related to factors such as training volume, training intensity, or, alternatively, the fact that several athletes conduct TMT as part of a resistance training program. Importantly, only studies examining TMT interventions were included, and all the studies were carefully screened for information on additional resistance training conducted [26]. Of the 31 included studies, only one study reported the performance of additional resistance training exercises conducted with an intensity of ≥ 50% of the 1RM [18]. However, the study included arm and shoulder muscles of the non-dominant arm in elite golfers. Still, unilateral resistance exercises have proven to increase the contralateral trunk muscles significantly in acute studies [8, 102]. Isolated versus integrated trunk exercises (e.g., compound lifts like deadlift and squat) is an on-going debate regarding TMT. Based on the methods described in included studies, the authors can neither reject nor support the proposal that present findings for effects of TMT in athletes may be attributable to, or affected by, other factors, and certainly debate is on-going as to the relative benefits of applying isolated versus integrated trunk exercises (e.g., compound actions, such as deadlift and squat) within TMT. A strength of the present investigation is that only studies that compared TMT with an active control group were included, to reduce the potential for regular training to bias outcomes in a sport-specific manner. Trunk adaptation, in response to sports-specific actions, is likely to vary according to activities regularly performed within a sports code; therefore the inclusion of non-TMT trained athletes, who otherwise trained at the same volume as their TMT counterparts, limits the potential for sport-dependent effects on trunk muscle adaptation to affect comparison between studies included in this paper.

### Limitations

This study has some limitations that need to be addressed. TMT is not a straight-forward concept involving homogeneous exercise programming, and can include trunk stability, trunk strength, trunk endurance, or a combination of these training types [4, 5, 15]. It is possible that differences in TMT methodology contributed to different effects on physical fitness outcomes, and this may explain the observed evidence of considerable heterogeneity found in the present analysis, which ranged from low to high (*I*^2^ = 28–81%), with > 75% rated as considerable [52]. In a sports-performance setting, TMT is never conducted in isolation but in combination with other training types (i.e., endurance, strength, power) and regular physical fitness training. The impact of TMT may therefore be blurred by adaptation attributable to other training types. For example, several studies have reported similar or greater trunk muscle activation during lower limb heavy resistance training than during isolated trunk exercises [24, 25, 103]. Nevertheless, the authors only included comparable studies in this review, which is a strength of the assessment of TMT on sport-specific and physical fitness outcomes. Even though the present review included double the number of studies compared with the previous meta-analysis by Prieske and colleagues [23], limited studies were found in elite athletes and in females. Additionally, the long-term effects of TMT on sport-specific performance are still unclear, given that mean intervention duration within included studies was only 9 weeks. Furthermore, none of the included studies examining adolescents reported maturation offsets, but only chronological age. Finally, it is recommended that future studies should clearly describe programming parameters such as number of sets and/or repetitions, intensity (using rate of perceived exertion), and session duration and frequency, in order to enable direct comparison of dose–response effects of TMT on performance outcomes in athletes. Given the potential for multidimensionality within TMT protocols, factors such as condition of prescription (use of external load vs. limb-load only) and emphasis on execution (muscle-specific vs. generalized trunk stiffness) require additional investigation, in order to assess whether determinants of TMT influence the effects on physical fitness and sport-specific performance in athletes.

## Conclusions

TMT is an important complement to sport-specific training in athletes. This meta-analysis found moderate effects for TMT on sport-specific performance, small-to-large effects on physical fitness, and moderate effects on trunk muscle endurance, when compared with non-TMT supplemented active controls. Potential moderator variables of TMT effects such as age, sex, and expertise level appear to have a minor effect on the overall outcomes. Based on our findings, we recommend that strength and conditioning coaches administer longer TMT training interventions (> 18 sessions) and/or employ short session durations (≤ 30 min) to improve lower limb muscle power, linear sprint speed, and CODS/agility. Longer sessions (> 30 min) at a frequency of twice per week appear more effective to improve sport-specific performance in athletes. Future TMT intervention studies are needed in elite athletes and females to increase our knowledge of TMT effects in elite performance populations and to investigate whether these vary in a sex-dependent manner. In addition, as TMT is not homogeneous and includes a variety of training modalities, further studies are needed to examine the effects of different TMT approaches on sport-specific parameters and physical performance outcomes.

## Supplementary Information

Below is the link to the electronic supplementary material.

Supplementary file1 (DOCX 22 kb)

## Declarations

### Data availability statement

The datasets generated and/or analyzed during the current study are available from the corresponding author upon reasonable request.
