# Supplementary material for: The Effects of Trunk Muscle Training on Physical Fitness and Sport-Specific Performance in Young and Adult Athletes: A Systematic Review and Meta-Analysis
Source: Sports Med. 2022 Jan 21;52(7):1599–622. doi: 10.1007/s40279-021-01637-0 (PMC9213339; doi:10.1007/s40279-021-01637-0)
Supplement: Supplementary file 1 — Supplementary file1 (DOCX 22 kb) [file 40279_2021_1637_MOESM1_ESM.docx]

Physiotherapy Evidence Database (PEDro) score for the included studies.

| Study | Eligibility criteria | Randomized allocation | Blinded allocation | Group homogeneity | Key out put  (< 15% drop out) | Intention-to-treat analysis | Between group comparison | Point to estimates and variability | Total PEDro score |
| --- | --- | --- | --- | --- | --- | --- | --- | --- | --- |
| Afyon, [63] |  |  |  |  | x | x | x | x | 4 |
| Afyon, [64] |  |  |  |  | x |  | x | x | 3 |
| Aslan et al., [68] |  | x | x | x | x | x | x | x | 7 |
| Bayrakadar and Boz, [76] |  |  |  | x | x | x | x | x | 5 |
| Butcher et al., [62] |  | x | x | x |  |  | x | x | 5 |
| Clark et al., [56] |  | x | x | x | x | x |  | x | 6 |
| Dağanay et al., [38] |  | x | x | x | x | x | x | x | 7 |
| Farhan et al., [75] | x | x | x | x | x | x | x | x | 7 |
| Fernandez-Fernandes et al., [72] | x | x | x | x | x | x | x | x | 7 |
| Genç and Ciğerci, [70] |  |  |  | x | x | x | x | x | 5 |
| Genc et al., [65] |  |  |  | x | x | x | x | x | 5 |
| Gencer, [69] |  |  |  | x | x | x | x | x | 5 |
| Hoshikawa et al., [71] |  |  |  | x | x | x | x | x | 5 |
| Hung et al., [57] |  | x | x | x |  |  | x | x | 5 |
| Karpiński et al., [60] |  | x | x | x | x | x | x | x | 7 |
| Kim, [59] |  |  |  | x | x |  | x | x | 4 |
| Kuhn et al., [53] | x | x | x | x | x | x | x | x | 7 |
| Manchado et al., [54] | x | x | x | x | x | x | x | x | 7 |
| Milles et al., [61] | x |  | x | x | x | x | x | x | 6 |
| Ozmen and Aydogmus, [73] |  | x | x | x | x | x | x | x | 7 |
| Ozmen et al., [74] | x | x | x | x | x | x | x | x | 7 |
| Panagoulis et al., [77] |  | x | x | x | x | x | x | x | 7 |
| Patil et al., [20] | x | x | x | x | x | x | x | x | 7 |
| Saeterbakken et al., [16] | x |  |  | x | x | x | x | x | 5 |
| Sato and Mokha, 2009 |  | x | x | x |  |  | x | x | 5 |
| Sharma et al., [55] | x | x | x | x | x | x | x | x | 7 |
| Sung et al., [18] | x | x | x | x | X | x | x | x | 7 |
| Taskin, [67] |  | x | x | x | x | x | x | x | 7 |
| Tse et al., [17] |  |  |  | x |  |  | x | x | 3 |
| Vigneshwaren, [66] |  | x |  | x | x | x | x | x | 6 |
| Weston et al., [19] |  |  |  | x | x | x | x | x | 5 |

X indicates a “yes” score. The eligibility criteria has been excluded from the total PEDro score.
